# Bio-Polyethylene (Bio-PE), Bio-Polypropylene (Bio-PP) and Bio-Poly(ethylene terephthalate) (Bio-PET): Recent Developments in Bio-Based Polymers Analogous to Petroleum-Derived Ones for Packaging and Engineering Applications

**DOI:** 10.3390/polym12081641

**Published:** 2020-07-23

**Authors:** Valentina Siracusa, Ignazio Blanco

**Affiliations:** 1 Department of Chemical Science (DSC), University of Catania, Viale A. Doria 6, 95125 Catania (CT), Italy; 2 Department of Civil Engineering and Architecture, University of Catania an UdR-Catania Consorzio INSTM, Viale Andrea Doria 6, 95125 Catania, Italy; iblanco@unict.it

**Keywords:** bio-based polymers, bio-based plastics, Bio-polyethylene (Bio-PE), bio-polypropylene (Bio-PP), Bio-poly(ethylene terephthalate) (Bio-PET), bio-polyolefins, packaging, food packaging

## Abstract

In recent year, there has been increasing concern about the growing amount of plastic waste coming from daily life. Different kinds of synthetic plastics are currently used for an extensive range of needs, but in order to reduce the impact of petroleum-based plastics and material waste, considerable attention has been focused on “green” plastics. In this paper, we present a broad review on the advances in the research and development of bio-based polymers analogous to petroleum-derived ones. The main interest for the development of bio-based materials is the strong public concern about waste, pollution and carbon footprint. The sustainability of those polymers, for general and specific applications, is driven by the great progress in the processing technologies that refine biomass feedstocks in order to obtain bio-based monomers that are used as building blocks. At the same time, thanks to the industrial progress, it is possible to obtain more versatile and specific chemical structures in order to synthetize polymers with ad-hoc tailored properties and functionalities, with engineering applications that include packaging but also durable and electronic goods. In particular, three types of polymers were described in this review: Bio-polyethylene (Bio-PE), bio-polypropylene (Bio-PP) and Bio-poly(ethylene terephthalate) (Bio-PET). The recent advances in their development in terms of processing technologies, product development and applications, as well as their advantages and disadvantages, are reported.

## 1. Introduction

**Figure 1:**
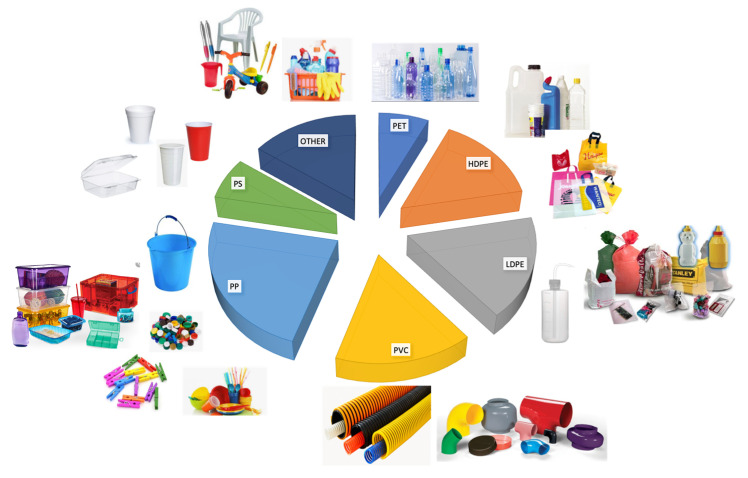
General distribution and application of plastics.

Today, the public interest in the environment, climate change and the limited resources of fossil fuel are driving governments, companies and scientists to find interesting and ecofriendly resource alternatives to petroleum. Polymers, that is to say plastics, that are obtained from petroleum are used for diverse and/or specific application, by tailoring their chemical structure well to the specific required performances. 85–90% are standard plastics, each one with its preferred application, but only seven types of polymers cover approximatively two thirds of the total plastics demand in all applications (packaging, building and construction, automotive, electric and electronics, others), and these are: Low Density Polyethylene (LDPE), Low Linear Density Polyethylene (LLDPE), High Density Polyethylene (HDPE), Polypropylene (PP), Polystyrene (PS), Polyvinyl chloride (PVC) and polyethylene terephthalate (PET) [1]. A general distribution of such commodity plastics is reported in Figure 1:

Polyolefins, followed by polyesters, comprise the major part of plastics, corresponding to about 45%, and are used especially for packaging applications, [1]. As still highlighted by Nguyen et al. [1], the depletion of 5% of petroleum and natural gas per year for their production, the continuous and dramatic rise in plastic consumption in view of the importance of considering the final destination of these materials (where are the 300+ billion kg of plastic produced each year?), and the interest in finding renewable resources to build our indispensable plastics must be followed. As an example, in the United States, 60% of plastics are landfilled, 10% are recycled and 30% are unaccounted for, contributing to terrestrial and aquatic environmental concern. Furthermore, as is also reported by Nguyen et al., the weight of plastic accumulated in the ocean will exceed the weight of fishes by 2050 probably, considering that 10 tons of PE, PP and foamed PS (floating plastics) are discarded every minute into the ocean. In this context, bio-based plastics could offer an important contribution to reducing the use of and dependence on limited petroleum resources and to decreasing the related environmental impacts. As reported by Niaounakis and by Shen et al. [2,3], bio-based plastics can be defined as “*man-made or man-processed organic macromolecules derived from biological resources and for plastic and fibre applications*”. They can be biodegradable or not, sometimes compostable and can be synthesized from renewable resources or from resources coming from plants and/or animals, and sometime they can return to nature as water, carbon dioxide, inorganic compounds and biomass via the composting process, leaving no toxic and/or distinguishable residues [4,5,6,7,8], according to ISO and ASTM rules. The biodegradability depends on the chemical structure of the polymers but not on the sources used for the monomers’ collection.

The bio-based plastic that has been investigated up to now could be divided into three principal groups [9]:

1. bio-plastics that are based on renewable resources and that are biodegradable, like starch plastic, cellulose polymers, proteins, lignin and chitosan plastics, polylactic acid (PLA), polyhydroxy alkanoates (PHAs), but also polyhydroxybutyrates (PHBs), polyhydroxyvalerate (PHV) and their copolymers in different percentages (PHBV); this class now includes polymers such as PVC, PE, PP, PET, nylon and polyamides (PA), named as bio-plastics because the starting monomers could be obtained from biological resources;
2. bio-plastics based on petroleum resources, which are 100% biodegradable, like polycaprolactone (PCL), polybutylene succinate (PBS), polybutylene adipate (PBA) and its copolymers with synthetic polyesters like polybutylene adipate-terephthalate (PBAT) and polyvinyl alcohol (PVOH);
3. bio-plastics obtained by using monomers coming from mixed biological and petroleum resources like polyesters obtained with petroleum-derived terephthalic acid and biologically derived ethanol, 1,4-butanediol and 1,3-propanediol, such as polybutylene terephthalate (PBT), polytrimethylene terephthalate (PTT), polyethylene-co-isorbite terephthalate (PEIT), polyurethane (PUR) and epoxy resins (thermoset plastic).

**Figure 2:**
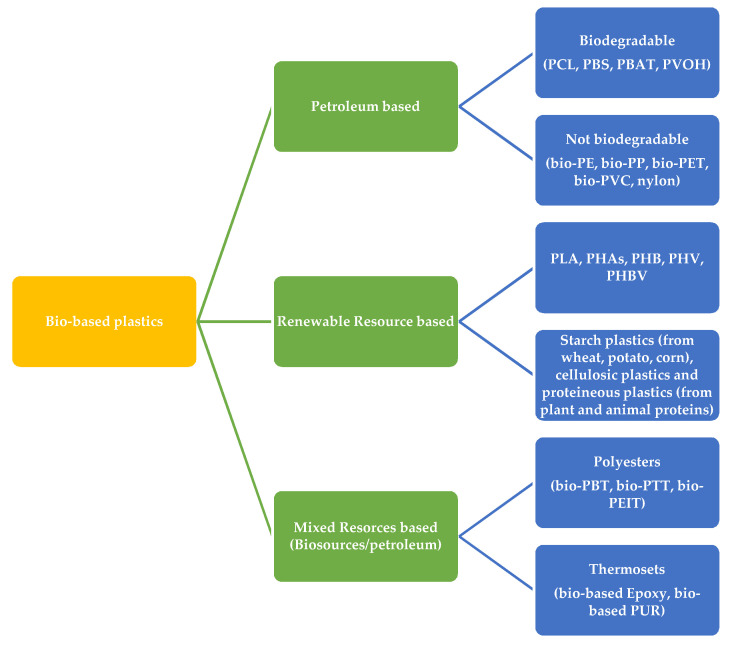
Classification of bio-based plastics.

A summarized personal overview of these plastics is reported in Figure 2:

Starting from 2000, bio-based plastics have attracted great interest thanks to the fact that in the last few years the development of new technological approaches has driven an interest in emerging new plastics obtained from renewable feedstocks [10,11,12,13]. Many old processes have been modified and revised in order to be used to produce chemical intermediates for polymer production, such as, for example, the chemical dehydration of ethanol, which could be used to produce Bio-PE and other plastics. Despite some of the plant capacities still being small when compared with the petrochemical ones, such as, for example, in the production of PHAs polymers, others are very big in size, such as the bio-based PE production plant. New materials with improved properties and special functionalities could be obtained thanks to the continuous advancements and innovations of the bioplastics industry [14,15]. Today, it is even almost possible to have a bio-plastic alternative to conventional plastic material, with nearly the same properties and performance but with the great advantage of reducing the carbon footprint or of being able to provide additional waste management options, such as industrial composting [9,16]. Furthermore, the growing concerns about the environmental impact of plastics and the interest to reduce the dependency on fossil fuel resources have increased the interest in such bio-plastics even more, with a steady increment in the number of materials, applications, products, manufacturers, converters and end-users. Regarding the economic, political and social development, this field is of great interest, especially for single-use plastics goods, the object of the Single-Use Plastics Directive implementation, of the new EU Commission’s European Green Deal of 2020, of the Circular Economy Action Plan and the framework for bio-based and biodegradable plastics [17,18,19]. A life cycle assessment (LCA) study could be a good tool to evaluate the environmental sustainability of materials and technology [20,21,22]. An interesting paper, presented by Chen et al. [23], reports a comparative LCA study on petroleum-based and bio-based PET bottles within the context of the United States. The consumer-use phase as well as the end-of-life phase (recycling and/or disposal) were excluded from the study, while the production phases were considered, from fossil resources and from biomass feedstock for PET and bio-PET, respectively. For the polymer production, fossil-based ethylene glycol (EG), fossil-based terephthalic acid (PTA), corn, switchgrass and wheat straw raw materials for bio-EG, and corn stover and forest residues for bio-PTA were selected as the building block materials. 12 PET bottles scenarios were established, and 1 kg of PET bottles was chosen as the functional unit [23]. The researchers found that biomass feedstock extraction and processing showed a greater impact in terms of environmental emission than the corresponding fossil processes did, due to the extra energy required for agricultural operations and due to the chemicals requested as fertilizers for the production. The results are highly sensitive to iso-butanol production [23].

Considering the emerging interest in the synthesis of traditional polymers by using biological low-cost resources and thanks to the advances in biotechnologies, in this review we have focused our attention on bio-plastics belonging to the first category, named Bio-PE, Bio-PP and Bio-PET, and we chose these because they are the most used polymers in the world, especially as packaging materials [24]. As reported in the literature, PE dominates milk packaging and cosmetic/detergent packaging, PP dominates many sub-segments but as minor player, and PET dominates the carbonated/non-carbonated drinks market [25,26].

## 2. Bioplastics Market and General Application

**Figure 3:**
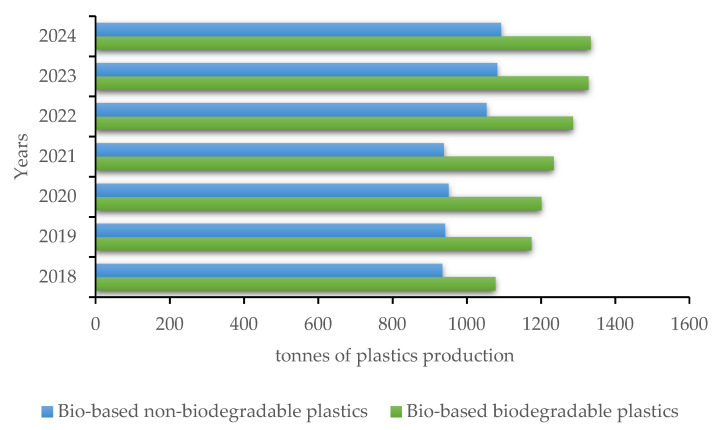
Forecast of the bioplastics production in tons from 2019 to 2024.

As reported by European Bioplastic [9], the total bioplastics production capacity is set to increase from around 2.11 million tons in 2019 to approximately 2.43 million tons in 2024, as reported in Figure 3:

Actually, bioplastic production represents about 1% of the 360 million tons of plastic produced annually in the world, but the market is in continuous increase thanks to even better technologies and emerging fields of interest and application [9]. Some polymers showed the highest growth rate, such as PHAs and bio-PP, with great industrial interest for their application in even more sectors. Thanks to this, the Bio-PP production is destined to grow by a factor of about six by 2024, while for the PHAs family the growth was estimated to be three times higher in five years, starting from 2019 [9].

**Figure 4:**
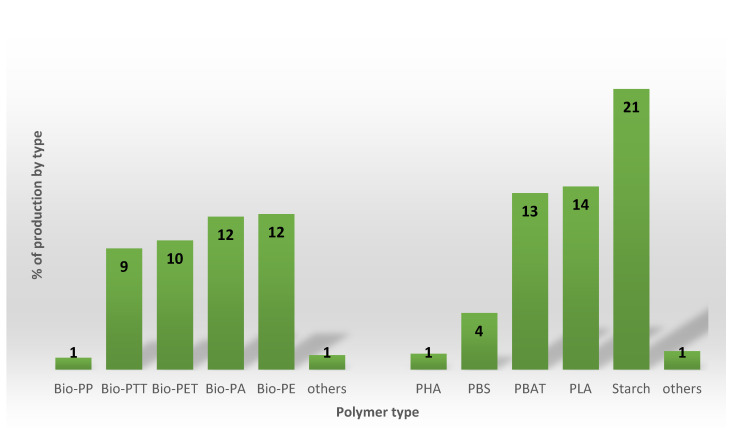
Percent (%) of global bio-based plastics production, by type.

**Figure 5:**
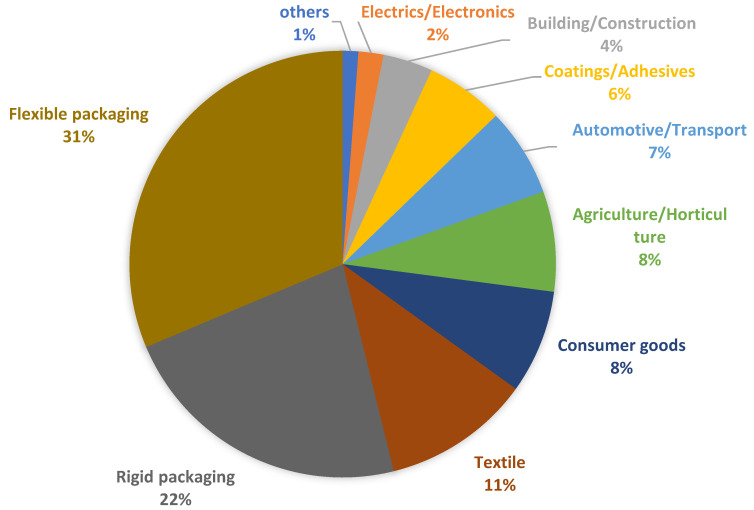
Market segments for bio-based plastics.

In particular, the global production capacities of bio-based non-biodegradable plastics account for about 45% of the total, while bio-based biodegradable plastics account for about 55% of the total, as following reported in Figure 4, by material type:

The largest field of application for bio-based plastics is packaging (both flexible and rigid packaging), which accounts for over 50% of 1.14 million tons of bio-based plastic production (in 2019), followed by textile, consumer goods, agriculture and horticulture, automotive and transport goods, coatings and adhesives, building and construction, electrics and electronics, and others like toys [9]. The percentage distribution is reported in Figure 5.

Asia is the major production hub in the world (45%), followed by Europe (25%), North America (18%) and South America (12%). The land used for the renewable feedstock for the production of bio-plastics actually comprises 0.016% of the total agricultural land, corresponding to 0.79 million hectares out of 4.8 billion hectares. 97% of those hectares are dedicated to pasture (3.3 billion hectares), feed and food (1.24 billion hectares), and others such as material uses (106 million hectares) and biofuels (53 million hectares). Despite the growing production of bio-plastics, in 2024 the land dedicated to them will reach a value of 0.021%, corresponding to 1.00 million hectares, highlighting even more the absence of competition between renewable feedstock used for the production of plastics and that used for food and feed [9].

## 3. Bio-Based Polyethylene (Bio-PE)

**Figure 6:**
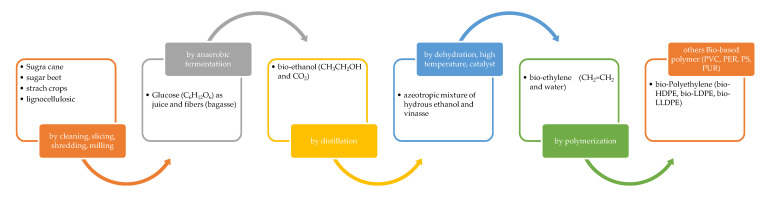
General scheme for Bio-PE production.

**Figure 7:**
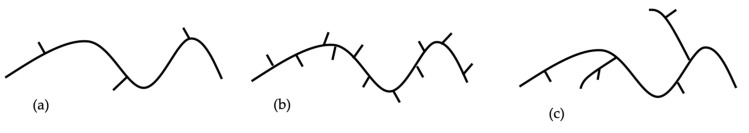
General chemical structure of (**a**) HDPE, (**b**) LLDPE and (**c**) LDPE polymers.

Thanks to its great performance, PE is the largest polymer used in the world. The starting monomer used for synthesis is ethylene, which is also the case for other polymers such as PVC and PS, and it is generally obtained from petroleum feedstock by distillation. However, great interest is now being shown to obtain this polymer from biological resources, and, as of now, the followed approach has been to synthetize the ethylene monomer by dehydration of bio-ethanol, obtained from glucose, following the general scheme reported in Figure 6:

The glucose could be obtained from different biological feedstocks, such as sugar cane, sugar beet, starch crops coming from maize, wheat or other grains and lignocellulosic materials. When starting from sugar cane, for example, processes like cleaning, slicing, shredding and milling are used to obtain the main sugar cane juice product and the sugar cane fiber by-product (bagasse). The juice, containing 12–13% of sucrose, is anaerobically fermented in order to obtain ethanol. The ethanol is distilled in order to remove water, giving an azeotropic solution of hydrous ethanol, at 95.5 vol.-%, and a by-product named vinasse. The subsequent polymerization of the ethylene monomer obtained this way is the same that is followed when using ethylene derived from petroleum, and the corresponding bio-polymer is identical in its chemical, physical, mechanical properties to fossil-based PE, also with regards to the mechanical recycling processes [27]. From polymerization, different types of bio-PE could be obtained: bio-HDPE with a low degree of short-chain branching (about seven branches per 1000 C atoms), bio-LLDPE with a high degree of short-chain branching and bio-LDPE with a high degree of short-chain branching + long-chain branching (about 60 branches per 1000 C atoms). For a better understanding of this, Figure 7 shows the chemical structures of such polymers:

All chemical, physical and mechanical characteristics are well reported in the literature, as are their engineering applications [28]. LLDPE is obtained by copolymerization of ethylene and butene, hexane or octane. For its processing, the same plant and machinery can be used [27]. Of course, bio-PE is not biodegradable. The bagasse by-product is used as a primary fuel source in the sugar mill process because its combustion produces an amount of heat that can cover the energy needs. A surplus of heat and/or electricity can also be produced, depending on the plant dimension. Vinasse by-product can instead be used as fertilizer [29].

From 2010, on a commercial scale, the first companies for the production of bio-PE have been the Brazilian company Braskem [30,31] for food packaging, cosmetics, personal care, automotive and toys applications (in this case in a joint venture with Brinquedos Estrela, a major Brazilian toy company), and the joint venture Dow and Crystalsev [32] for food packaging, as well as agricultural and industrial purposes. Dow is the second largest chemical manufacturer in the world, while Crystalsev is the major Brazilian ethanol producer. Their joint venture was born in order to produce bio-LLDPE. Furthermore, the chemical companies Solvay, Nova Chemicals and Petrobras are in the bio-PE market, contributing to the production of about 20% of the world’s bio-based plastics production. Braskem will cover 10% of the worldwide plastic market by the year 2020 [32].

At the beginning, bio-ethylene production was not considered to be cost-competitive when compared with petrochemical-derived ethylene, but starting from 2008 the price of one barrel of ethanol derived from sugar cane has become competitive with the price of one barrel of crude oil (about $115 US versus $80 US; 1 L = 0.0085 barrel). The price of 1 kg of bio-PE is about 30% higher than petrochemical PE [32].

Actually, as mentioned before, bio-ethylene derived from bio-ethanol is used for the synthesis of other polymers like PVC (used in the construction and building industry), due to severe criticism concerning impacts on the environment, human health and safety, especially in the packaging field, due to the difficulty of separating PVC from post-consumer waste. PS, epoxy resins and rubbers like ethylene propylene diene monomer rubber (EPDM) could be further bio-based candidates for the use of bio-based ethylene. Enriquez et al. [33] reported a study where Bio-PE (in particular Bio-HDPE) was used in blends with bio-PTT (37% bio-based) in order to obtain a copolymer with a good balance between stiffness and toughness. Bio-PE, with the trade name SHC7260, was produced by Braskem company (Brazil) and provided by Competitive Green Technologies (Ontario, Canada) and was used as received. Blends were mechanically obtained by melt processing in a co-rotating twin screw extruder and then injected in a mold at 30 °C. Different PPT/Bio-PE blends were obtained (0, 20, 40, 50, 60, 80, 100 wt.% of Bio-PE). From thermal analyses via differential scanning calorimetry, immiscibility was observed due to the presence of individual melting peaks at 230 °C for PPT and 118 °C for Bio-PE, showing a poor interfacial adhesion with a decreasing stiffness [33].

Brito et al. [34] investigated the mechanical performance of PLA/Bio-PE blends, while Castro et al. [35] and Kuciel at al. [36] investigated the performance of Bio-PE after the addition of natural fibers, used to increase the stiffness of the resulting materials. In particular, Kuciel et al. explored the mechanical performance of bio-composite materials obtained with bio-PE and synthetized using ethanol coming from sugarcane (Braskem, Brazil) and four different natural fillers (wood flour, ultrafine cellulose powder, kenaf chopper fibers and microparticles of mineral tuff filler) at a percentage of 25%, in comparison to net bio-PE. These composites were obtained via compounding extrusion molding followed by injection molding. Natural fibers and particles, with a low environmental impact and low cost, were used in order to improve the mechanical strength and modulus [37,38,39], while bio-PE was chosen as a bio-based polyolefin green matrix for structural Natural Fibre Composites (NFC) and Wood Plastic Composites (WPC). The disadvantage of using such fillers for a structural application is that PE-based WPC or NFC becomes hygroscopic if in contact with moisture and a water environment, leading to dimensional changes and accelerated ageing [36]. This behavior is attributed to a water-swollen filler, as well as the degradation of the fillers and of the interfacial adhesion between the filler and the polyolefin matrix. The use of fine cellulose microparticles and tuff microparticles, which are the easiest to process, showed a higher elongation at break and a lower water uptake.

## 4. Bio-Based Polypropylene (Bio-PP)

**Figure 8:**
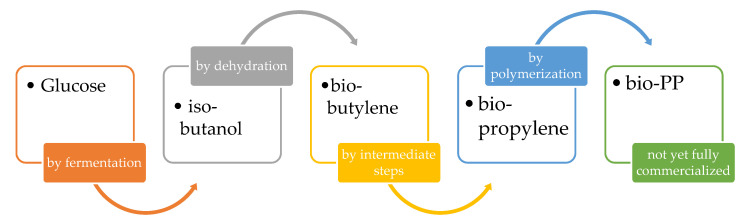
General scheme for Bio-PP.

After ethylene, propylene is the most important organic building block for poly-olefin production. PP is the second most important polymer after PE [40]. Bio-PP could be obtained from biological resources by butylene dehydration of bio-isobutanol obtained from glucose and subsequent polymerization, following the general scheme reported in Figure 8:

With respect to the production of Bio-PE, the process followed to obtain bio-PP has been less explored, which explains why bio-PP has not yet been commercialized. As for bio-PE, the first company that produced Bio-PP on a pilot-plant scale was Braskem, but their process route is still confidential. The most promising route to obtain propylene is probably through methanol, further processed to obtain propylene monomer via Lurgi’s methanol-to-propylene (MTP) process or UOP’s methanol-to-olefins process [32], using the same industrial plants as for petrochemical methanol.

As reported by Chen and Patel [41], another route could be the production of 1,2-propanediol or acetone through fermentation and a further conversion to 2-propanol by dehydration to propylene.

## 5. Bio-Based Polyethylene Terephthalate (Bio-PET)

Polyesters represent a large group of polymers that have the potential to be produced from bio-based feedstocks [42,43]. The most relevant for a shift towards bio-based chemicals are PET, PBT, PBS, PBA and the copolymers PBAT, poly(butylene succinate-co-lactate) (PBSL), poly(butylene succinate adipate) (PBSA) and poly(butylene succinate terephthalate) (PBST), but also polyvinylacetate (PVAc), polyacrylates, poly(trimethylene naphthalate) (PTN), poly(trimethylene isophthalate) (PTI) and thermoplastic polyester elastomer, as reported by the European Bioplastic Report [9]. Those polymers are produced from a bio-based diol, while diacid or diester could be either bio-based (like succinic or adipic acid) or petrochemical-based (like PTA and/or di-methyl terephthalate, DMT).

**Figure 9:**
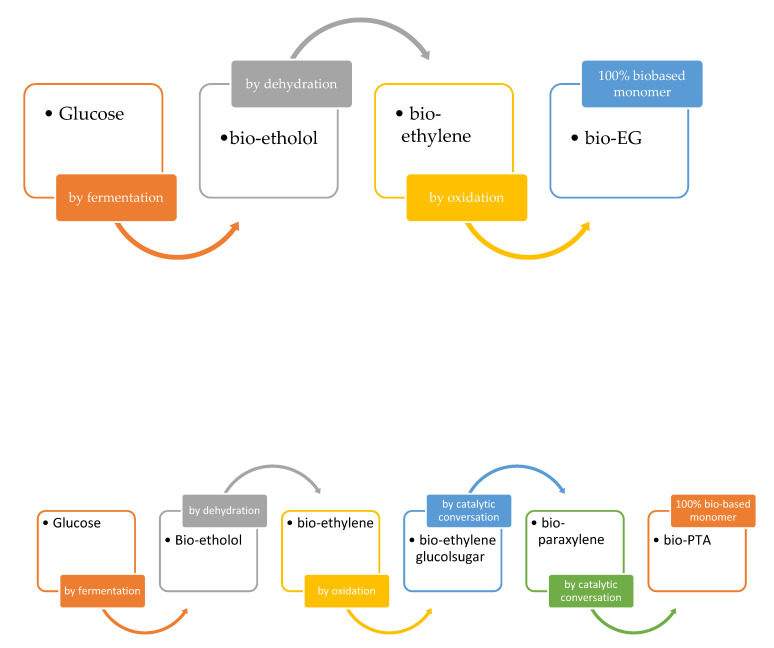
General scheme for Bio-EG and Bio-PTA monomers production.

Among them, PET is the largest used polyester, with physical and mechanical properties that make it suitable to be used for fibers (65%) and packaging (35%) applications. This last application regards bottles (76%), containers (11%) and films (13%). It plays an important role in the plastic market, but due to its poor sustainability due to a much slower degradability, it poses serious environmental issues, especially for waste treatment, when it is used for short time applications, as for example when it is employed as food packaging. It was first commercialized in 1940, especially for synthetic fibers and film applications. From 1970 onwards, it was used to produce bottles, with a continuous growing development. Since 2010, in order to realize a sustainable plastic, both starting chemicals such as ethylene glycol (EG) and PTA and/or DMT monomers have to be obtained from biological sources [44,45,46,47]. The general scheme of synthesis is reported in Figure 9:

The aliphatic monomer Bio-EG could be synthetized from the hydrolysis of ethylene oxide, obtained via the oxidization of bio-ethylene obtained from the fermentation of glucose, followed by dehydration [48,49]. Another route could be via sorbitol, based on hydrogenolysis [32,50]. The percentage of bio-based monomers can be varied, according to the stoichiometry of the reaction, obtaining a partial bio-based polymer. In 2009, the Coca-Cola Company introduced a 30% bio-based beverage bottle, named “PlantBottle”, made of 100% bio-based EG and petroleum-derived TA [51]. Toyota Tsusho Corporation, Japane Future Polyesters, Coca-Cola—Gevo Venture, PepsiCo-Virent Venture are actually the producers of Bio-PET [45]. Another route to synthetize EG from biomass is that proposed by Salvador et al. [52], via the use of different types of microorganisms. Among them, the bio-synthesis of EG could be achieved in bacteria, in high yields, by a pentose pathway, using xylose as a substrate. As reported by them, “*xylose is first transformed into xylonate by the action of a dehydrogenase; after the subsequent action of a dehydratase and an aldolase, glycoaldehyde is obtained, which is finally reduced to EG by a reductase*”. This procedure, with a yield of 98%, could be considered a promising alternative for the synthesis of bio-EG. Other approaches, such as through glucose in *Saccharomyces cerevisiae* using glycolytic enzymes [53], through the synthesis of serine in an engineered pathway in *E. coli* [54], using synthesis gas through the Wood–Ljungdahl pathway of carbon fixation present in acetogenic bacterial species such as *Moorella thermoacetica* and *Clostridium ljungdahlii* [55], and through gaseous alkenes using a strain of *E. coli* [56] could be used in a similar way.

**Figure 10:**
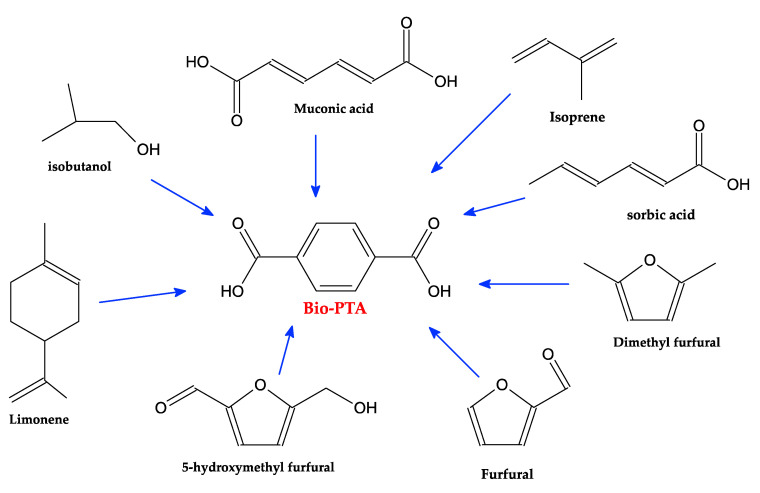
Schematic representation of the methods used to achieve Bio-PTA.

Following a different route, but always starting from biomass feedstock, a bio-PTA could also be synthetized, as schematically reported in Figure 8. In this case, different methods could be followed: the iso-butanol method, the muconic acid method, the limonene method or the furfural method [46]. The iso-butanol method was developed by Gevo, starting from sugar, which is considered a key building block for different chemicals such as isooctane, ethyl *tert*-butyl ether, methyl methacrylate and other alkanes. Following this method, p-xylene is first produced by the cyclization of two isooctane molecules, via the dehydrogenation route; second, via oxidation, the *p*-xylene is converted to TA. Carraher et al. and Matthiesen et al. [57,58] proposed a route starting from *cis,cis*-Muconic acid as the starting building block, obtained from sugar (through a combination of chemical processes and biorefining). After a sequence of *cis-cis* to *trans-trans* transition steps of muconic acid, a tetrahydro terephthalic acid (THTA) is obtained by ethylene addiction reactions, and, subsequently, bio-PTA is obtained by dehydrogenation. Colonna et al. [59] started the synthesis of terephthalate polyesters from limonene. As a starting building block, p-cymene was used, obtained by the chemical refining of limonene. They reached an 85% conversion from limonene by the oxidation of the iso-propyl group with potassium permanganate in concentrated nitric acid. Several researchers considered the possibility of converting furan derivate chemicals into PTA monomer (furfural method) [60,61,62,63,64] via the well-known Diels–Alder chemical reaction. First, furfural or other furan derivatives obtained by an inedible cellulose biomass is converted to maleic anhydride by oxidization and dehydration reactions; maleic anhydride is then involved in the reaction with furan in order to obtain a Diels–Alder adduct. By the subsequent dehydration of the adduct, a phthalic anhydride is obtained, finally converted into bio-PTA through phthalic acid and dipotassium phthalate. The process is still not industrialized. Tachibana et al. [65] also measured the bio-based carbon content through a mass spectroscopy analysis, measuring the ratios of the three carbon isotopes (^14^C, ^13^C, ^12^C). The proposed methods are schematically shown in Figure 10:

An interesting advantage of furfural is that it can be easily converted into other chemicals used for the synthesis of bio-based polymers, such as PBS and polyoxabicyclates [65].

Another interesting result was presented by Avantium, the developer of the bio-based furandicarboxylic acid (FDCA). Starting from fructose, they obtained a hydroxymethylfurfural chemical molecule (HMF), which was converted to dimethyl furane (DMF) by a hydrogenation reaction. Then, after several chemical steps, *p*-xylene was obtained and subsequently converted to bio-PTA, as previously described. Schenk et al. [66] reported about the BioBTX pilot plant, which is used to produce aromatic molecules such as benzene, toluene and terephthalate by means of biomass catalytic pyrolysis (wood and lignin-rich resources).

Following research by Chen et Patel [41], the aromatic monomer terephthalic acid or dimethyl terephthalate ester could be produced using lignin, the most abundant bio-renewable resource, which can be found in all vascular plants and which is the second most abundant organic polymer [67]. The aromatic aldehydes vanillin and syringaldehyde could be obtained by lignin extraction in order to further obtain bio-based p-xylene and then subsequently convert it into terephthalic acid.

The bio-based PET obtained this way can be processed by injection molding, blow molding and extrusion, and it is identical to petrochemical PET. Of course, it is not biodegradable, but an interesting paper, presented by Salvador et al., described a microbial degradation of PET due to the action of microbial polyester hydrolase, considered a key alternative for recycling PET [52]. Actually, the glycolysis method is advantageous for depolymerizing opaque and colored PET that cannot be recycled due to the presence of pigments, but the high energy cost associated with the high temperature required and the long reaction times needed for depolymerization make this process not economic and environmentally sustainable. The resulting EG and PTA monomers could be reused in the synthesis of PET, as well as for other polymers [68]. As an alternative to chemical PET depolymerization methods, a series of enzymes coming from different microorganisms could be used for a more sustainable strategy.

An interesting method to determine the amount of biogenic fraction was presented by Jou et al. [69] in their short communication on the use of Radiocarbon Accelerator Mass Spectroscopy for the isotopic fraction determination, according to ASTM-D6866 (Standard Test Methods for Determining the Biobased Content of Solid, Liquid, and Gaseous Samples Using Radiocarbon Analysis). As reported by Montava-Jorda et al. [70], bio-based polymers, either biodegradable or non-biodegradable, are certified as bio-based according to international standards such as EN 16640:2015, ISO 16620-4:2016, ASTM 6866-18 and EN 16785-1:2015. In particular, EN 16640:2015, ISO 16620-4:2016 and ASTM 6866-18 measure the bio-based carbon content in a material through ^14^C measurements, while EN 16785-1:2015 measures the bio-based content of a material using radiocarbon and elemental analyses.

An alternative approach is to replace terephthalic acid by different compounds derived from biomass, such as 2,5-furandicarboxylic acid (FDCA) for the production of polyethylene furanoate (PEF), which is not the object of this review [71,72,73].

## 6. Discussions

The use of bio-feedstock in the polymer sector is of great interest. Starch, cellulose, but also alkyd resins and some polyamides have been obtained from natural resources on an industrial scale, with an estimated worldwide production of 20 Mt/year (7% of the total plastics production). In contrast, the production of bio-based materials is estimated to be less than 2% of the total plastic production, despite the industry having demonstrated a great ability to process large amounts of biomass. Nevertheless, it is difficult to estimate the amount of such biomass that is necessary to replace petrochemical polymers by bio-based ones and whether this substitution would lower the environmental footprint. From a technological point of view, the progress in the plants processes have been spectacular. Some plants still have a rather small capacity, but others are comparable to typical petrochemical plants, like Braskem’s bio-based PE plant. With the continuous growing demand for bio-based plastics, plants with a bigger capacity production will be even more commercially available. From a geographic point of view, the United States and Europe are the most important areas for bio-based plastic production, followed by the Asia Pacific region and South America, with important investments [9].

**Figure 11:**
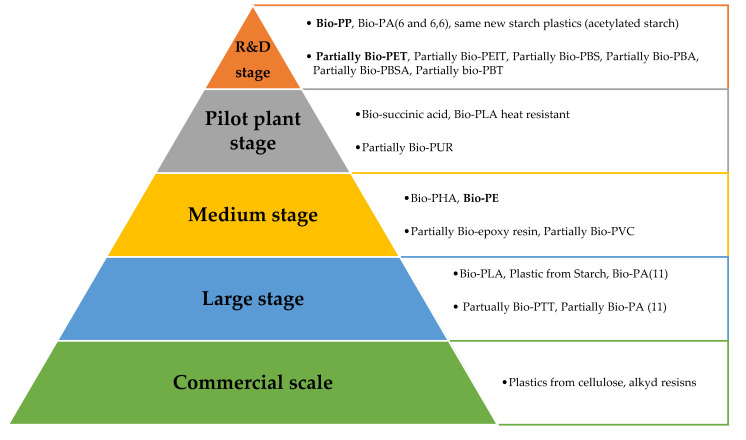
Development stages for emerging bio-based polymers.

Figure 11 shows the actual development stage of emerging bio-based polymers:

As can be observed, the largest number of bio-based plastics are in the R&D and pilot plant stage, while only few materials are in large and commercial stages. In the first case, different kinds of technological challenges are only possible. For the production of partial Bio-PBS, no technological barriers are present, because the production of succinic acid is technically ready. The esterification of succinic acid with butanol to obtain PBS is performed at a large scale but via petrochemical precursors. With respect to PBS, the production of partial Bio-PET is even more easy. Meanwhile, the production of Bio-PP requires several steps that present limited knowledge, making the scale-up even more difficult and more demanding. Thus, depending on the type of plastics, between 20% and 100% of the actual volume could be replaced by bio-based alternatives, but not in the short/medium term due to economic problems related to cost production and capital availability, technical scale-up, the great amounts of bio-based feedstocks and the industrial conversion to the new plastics. It has been estimated that from 2030 onwards, the substitution of petrochemical plastics with bio-based plastics is expected to be even higher, thanks to the replacement of the monomers by chemically identical bio-based compounds or bio-based compounds with the same equivalent functionality. However, if the technology becomes ready in plants, there are still several factors that can influence the speed of commercialization: financial, technology, personnel involved, interaction with other sectors, collaboration with other plants and companies from the agroindustry chain, market concerns related to the demand for bio-based goods by retailers and producers of consumer products, and, finally, regulation factors related to fiscal policy measures and public procurement.

Another important factor to take into consideration in the production of bio-based materials is the competition with food, feed and bio-fuels for the supply of raw materials. Today, starch and sugar crops are the sole feedstocks supply, but a second generation of bio-ethanol, based on lignocellulosic feedstocks, can be used as future starting chemicals in the production of bio-PE, as well as for other biopolymers (like PLA) [74].

Lignocellulose is cheap, abundant and not competitive with food with respect to starch and sugar crops, but the biotechnology to transform cellulose into sugar monomers, via microorganisms, requires processing steps that are more complicated. For the next two decades, the biomass demand for bio-based plastics production will remain smaller than that demanded for human food and animal feed.

The major influencing factors for the development of bio-based plastics are the technological barriers that must be overcome in order to reach a large-scale production, the suitability of these materials to be used as bulk materials, their competitiveness in terms of cost with respect to petroleum-derived ones and the availability of raw materials for their synthesis. A very interesting paper is that of Volanti et al. [75], which reports a sustainable analysis used to estimate the environmental performance of three routes followed to obtain bio-PTA for the synthesis of bio-PET: from sweet corn, from sugar beet and maize grain, and from orange peels. The results were compared with traditional technology. The production using sugar beet and maize grain was closest to the current fossil technology, the production with sweet corn was found to be the most impactful, while the production using orange peels was the greenest one. The researchers found that the bio-routes for synthetizing PTA could only be very competitive if organic waste streams were converted into raw materials for the production of building blocks; however, if dedicated crops were used, some limitations were observed on the mitigation of climate change. From this research, it is clear that an early stage evaluation is fundamental in order to identify the benefits and the disadvantages of bio-based plastic production and commercialization. It was pointed out that an important impact is recorded when crops are used, even when compared to fossil fuels use. Meanwhile, waste is a very interesting and valuable building blocks resource, showing two advantages: first, wastes are available while biomass requires dedicated cultivation, and, second, there are economical and environmental savings that result from the non-disposal of waste itself.

The potentiality of these bio-based polymers to be used in bulk applications is not a problem, thanks to the fact that they are chemically identical with their petrochemical counterparts. Within the bio-based plastics sector, PLA, bio-PE, starch plastics and bio-based epoxy resins are the four key materials. PLA and starch plastics are candidates for fast growth thanks to their production cost reduction and bulk application. Bio-based epoxy resins will only grow if the bio-based glycerol starting monomer is available and its costs are lowered. Meanwhile, the key factor for the growth of Bio-PE is related to its production costs. Bio-PP is still in its lab/pilot-plant stage due to technological barriers that include conversion technologies and downstream processing technology. When considering an optimistic future scenario, it is expected that it will leave the pilot stage and enter the commercialization stage from 2021 onwards.

## 7. Conclusions

Bio-based plastics represent a very interesting and emerging field, and the even larger growth in the use of biomass feedstock for non-food purposes has demonstrated the technical possibility of producing these materials on a million-ton scale, substituting petrochemical plastics in meaningful quantities. Actually, the most important polymers in terms of production volumes are PLA and starch plastics, but the intended trend is to promote and increase the world-wide capacity of different types of bio-based plastics. In view of the most important and representative company announcements, bio-based PE and PHA, will also become as important as PLA and starch plastics by this year.

Since 1980, interest in bio-based plastics, especially biodegradable plastics, has been driven by ever more sizeable waste management problems, but now much more attention is being focused on durable plastics as a means of reducing wastes. The interest is to take advantage of the use of biomass coming from food and agricultural wastes, choosing monomers and polymers that can be easily processed within existing structures. Ethylene and propylene are a great example of this interest. In the next decade, the interest in such bio-based durable plastics will grow much more than bio-based biodegradable plastics, partly due to the fact that the management of bio-based biodegradable plastics requires major changes and investments in the waste management infrastructure with respect to petroleum-like polymers. Nevertheless, bio-based biodegradable plastics will grow continuously, occupying their own position in the plastic market.

Small and medium enterprises (SMEs), such as Novamont, Biotec, Rodemburg Biopolymers, Cereplast, Tianan and so on, as well as large chemical companies, such as Braskem and Dow, are very active in the field of bio-based plastics. Actually, for big companies, many projects on bio-based polymers represent a relatively small share of their interests, but in a near future they will have the opportunity to convert their production in order to avoid environmental impact problems, such as CO_2_ emissions, non-renewable energy saving, greenhouse gas reduction, and waste reduction and management. Despite the existence of numerous plastic materials with a high bio-renewability, only a small fraction of these have found a place in commercial applications. The heaviest challenges will be to reduce the high cost of production and processing, minimize agricultural land use and forests, avoid competition with food production, and, of course, reduce the environmental impacts.
